# The effect of genotype and muscle type on the physico-chemical characteristics and taurine, carnosine and L-carnitine concentration in lamb meat

**DOI:** 10.5194/aab-63-423-2020

**Published:** 2020-11-26

**Authors:** Aurelia Radzik-Rant, Witold Rant, Gabriela Sosnowiec, Marcin Świątek, Roman Niżnikowski, Żaneta Szymańska

**Affiliations:** Department of Animal Breeding, Institute of Animal Sciences, Warsaw University of Life Sciences-SGGW, Ciszewskiego St. 8, 02-786 Warsaw, Poland

## Abstract

An experiment was conducted to determine the chosen bioactive components and physico-chemical characteristics of lamb meat of different animal genotypes and the muscle types. The 22 ram lambs of Polish Merino (PM) and 22 crossbreeds of Polish Merino × Berrichone du Cher (PMB) were fattened to achieve their slaughter weight of 40 kg. After slaughter, the carcasses were kept at 4 ${}^{\circ }$C for 24 h. Then, the samples of *longissimus lumborum* (LL) and *gluteus medius* (GM) muscle were collected to analyse the physico-chemical traits; fatty acid profile; and concentrations of taurine, carnosine, L-carnitine. The GM muscle compared to LL had the higher value (P < 0.05) of $L^{*}$ and a lower value (P < 0.05) of $b^{*}$ and $H^{*}$ both in PM and PMB lambs. The value of expressed juice was lower (P < 0.05) in both LL and GM muscles of PM lambs. A higher amount (P < 0.05) of collagen was found in LL muscle compared to GM both in PM and PMB lambs. The GM muscle of PM lambs showed higher (P < 0.05) conjugated linoleic acid (CLA) content, as well as higher total polyunsaturated acids (PUFAs), PUFA n-6, and PUFA n-3 (P < 0.05). The GM muscle was characterized by a higher (P < 0.05) content of taurine, while in the LL muscle there was a higher amount (P < 0.05) of carnosine. A larger amount (P < 0.05) of L-carnitine was found in GM muscle but only within PMB lambs. The obtained results showed a greater impact of the lamb's genotype on the physical characteristics of meat than on its chemical composition and the content of bioactive components. The muscle type had an effect on meat colour; collagen content; fatty acid profile; and amount of taurine, carnosine, and L-carnitine present.

## 1 Introduction

Meat is one of the basic component of the human diet and contains nutrients that guarantee the proper development of the organism and prevents the occurrence of many diseases resulting from their deficiency (Schmid, 2010). It is an important source of valuable and easily digestible proteins, as well as many minerals and vitamins. Bioactive components found specifically in red meat allow it to be included as a functional food (Decker and Park, 2010). These ingredients include, among others, taurine, carnosine, anserine, L-carnitine, and conjugated linoleic acid (CLA). All of these compounds play an extremely important role in the human body, they have antioxidant, buffering, regulating, or anti-cancer properties and are not always synthesized in sufficient quantity. Their required levels are often provided by a proper diet (Mora et al., 2008; Schmid, 2010; Yang et al., 2015).

A number of factors influence the content of biologically active ingredients beneficial for human health, as well as the physico-chemical characteristics of meat, which are important for the consumer in terms of culinary value. These features depend on the animal species, breed or type of crossbreeding, maintenance system, diet, and the type of muscle (Purchas et al., 2004; Knüttel-Gustavsen and Harmeyer, 2007; Peraza-Mercado et al., 2010).

The development of different types of muscle fibres affects not only the overall muscle mass of slaughter animals but also their physico-chemical characteristics (Lee et al., 2010), whereas the animal's genotype is a factor that affects the physico-chemical characteristics of meat to a lesser degree (Carson et al., 2001; Peraza-Mercado et al., 2010; Radzik-Rant et al., 2014). However, the genotype may affect the proportion of biologically active compounds in meat. The study conducted by Peiretti et al. (2011) has shown that ruminant's meat contains more bioactive protein components than meat of other species. Lamb meat, especially of breeds of lower fatness, is distinguished by a higher content of biologically active lipids (Wood et al., 2008). The level of these compounds may also depend on the muscle and different morphological, metabolic, and functional characteristics of fibre types they contain (Knüttel-Gustavsen and Harmeyer, 2007; Schmid, 2010; Jayasena et al., 2014).

The aim of this study was to analyse the chosen bioactive components and physico-chemical characteristics of lamb meat, taking into account the animal's genotype and the type of muscle.

## 2 Material and methods

Research on animals was conducted according to the institutional committee on animal use (Second Local Ethical Commission for Animal Experimentation in Warsaw, consent form no. WAW2_20/2016).

This experiment was carried out on 22 ram lambs of the Polish Merino (PM) breed and 22 crossbred lambs of Polish Merino × Berrichone du Cher (PMB). The lambs were fattened to achieve their slaughter weight of 40 kg (± 1.5 kg). The animals were kept in a barn on straw bedding under uniform environmental conditions with constant zootechnical and veterinary supervision.

The lambs were fed in a group according to the standards for fattening lambs (Osikowski et al., 1998). The ration consisted of grass hay; steamed potatoes; and a concentrate containing 57.8 % oatmeal, 17.6 % wheat bran, 23,5 % rapeseed meal, and 1 % mineral mixture. The chemical composition and nutritional value of the fodder are presented in Table 1. The animals were fed twice a day. The grass hay was fed to the lambs separately. The potatoes and concentrate were mixed before each feeding. The animals had constant access to water.

**Table 1:** The chemical composition and nutritional value of feeds used in lambs fattening.

Component	Grass	Oat	Rapeseed	Wheat	Steamed
	hay	meal	meal	bran	potatoes
Dry matter (g kg${}^{-1}$ )	870.4	880.8	902.1	887.3	192.8
Crude protein (g kg${}^{-1}$ DM)	129.8	97.7	325.5	134.6	115.0
Ether extract (g kg${}^{-1}$ DM)	22.0	31.8	34.8	31.6	3.0
Crude fibre (g kg${}^{-1}$ DM)	317.0	90.5	104.6	62.8	37.0
Ash (g kg${}^{-1}$ DM)	36.5	22.6	67.6	43.7	52.9
EN (MJ kg${}^{-1}$ DM)	3.72	6.56	7.47	5.87	8.87

EN: net energy; MJ: megajoule; DM: dry matter.

After reaching the desired body weight, lambs were slaughtered according to Council Regulation (EC) no. 1099/2009 of 24 September 2009 (EU Acts Office dated 18 November 2009 L 303/1). Carcasses were suspended from the Achilles tendon and chilled at 4 ${}^{\circ }$C for 24 h.

### 2.1 Sample preparation

From the right side of each carcass, the samples of *longissimus lumborum* (LL; n=44) and *gluteus medius* (GM; n=44) muscle were collected and vacuum packed. The samples were transported in the refrigerator to the laboratory in order to perform quality analysis.

Samples of approximately 700 mg were taken from the middle part of each muscle, fixed in liquid nitrogen, and then stored at -80 ${}^{\circ }$C until analysis of the concentration of taurine, carnosine, and L-carnitine. The rest of the LL and GM muscle samples were used to determine physico-chemical characteristics of the meat.

### 2.2 Analysis of physical traits of meat

The pH was measured 24 h after slaughter using an Elmetron CP-411 pH meter with a dagger electrode calibrated at pH values of 4.0, 7.0, and 9.0.

The colour of LL and GM samples was measured on the meat surface, after 30 min of blooming, using a Minolta CR-410 (Konica-Minolta) colourimeter. The following colour coordinates were determined: lightness ($L^{*}$), redness ($a^{*}$) and yellowness ($b^{*}$), colour saturation ($C^{*}$), and hue ($H^{*}$). Measurements were performed three times for each sample.

The expressed juice was determined by the Grau and Hamm (1953) method. Samples (0.3 g) of meat were placed on Whatman filter paper no. 1 and held under a pressure of 2 kg for 5 min. The outline area of the expressible juice and the meat film was traced, and two areas were measured using a planimeter. The results have been calculated in units of square centimetres per gram of meat.

### 2.3 Chemical composition of the meat

The components of moisture, crude protein, intramuscular fat, and collagen were determined using a spectrometric technique with a near-infrared transmission (NIR) method (PN-A-82109). The meat samples were homogenized in an Elektrolux DITO K35 processor. Following this, unified samples were placed in a measuring cell of a FoodScan analyser. The device uses the near-infrared transmission method within 850–1050 nm range and is fitted with ANN calibration developed using a model of artificial neural networks. The analysis is performed by indicating the number of 16 measurements in the sample in the computer program, and then the program automatically calculates the average and presents the result.

### 2.4 Fatty acid determination

To extract the lipids from the meat samples the method of Folch et al. (1957) has been used. The fat saponification was performed in 0.5 M potassium hydroxide (KOH) in methanol, and esterification was performed in 10 % BF3 in methanol. The fatty acid methyl esters were extracted in the hexane.

The analysis of the fatty acid profile was performed by a gas chromatograph using the Agilent Technologies GC 6890 N device equipped with capillary column BP × 70 (length 60 m, internal diameter 0.22 mm, film thickness 0.25 µm). Operational conditions included helium gas (41 psi) and an FID (flame ionization detector) at 240 ${}^{\circ }$C. The temperature program was 3 min at 130 ${}^{\circ }$C, an increase to 235 ${}^{\circ }$C by +2 ${}^{\circ }$C min${}^{-1}$, and 4 min at 235 ${}^{\circ }$C.

The fatty acids were identified via reference material BCR 163 (beef and pig fat blend). The isomer of linoleic acid (CLA) was determined by standard *cis-9, trans-11* octadecadienoic acid (Larodon AB, Sweden).

### 2.5 Taurine, carnosine, and L-carnitine analysis

To determine the taurine, carnosine, and L-carnitine concentration in meat samples the ELISA immunoassay test has been used. For each meat sample previously stored at -80 ${}^{\circ }$C, triplicate 250 mg samples were homogenized in 25 mL PBS (phosphate-buffered saline). After that, the homogenates were centrifuged for 5 min at 5000 × g on ice. The supernatants were assayed using Enzyme-linked Immunosorbent Assay Kits according to manufacturer's protocols (CLOUD-CLONE Corp., USA).

A microplate reader WHY101TX (Novazym Poland Ltd.) was used for spectrophotometric reading of the results. The concentration of taurine, carnosine, and L-carnitine was calculated from the determined standard curves for each sample.

### 2.6 Statistical analysis

A statistical analysis of the data was performed using the SPSS 23.0 packet software (2016) based on a linear model that included the effect of genotype, type of muscle, and the interaction between genotype and muscle type. All effects were tested against residual middle squares to determine the level of significance. Tukey's test was used for comparing mean values when an F test for main effect was significant. The results are presented as the least-squares means (LSMs) for each trait and standard error (SE).

## 3 Results

### 3.1 Physical characteristics of the meat

There were no statistical differences in the pH either between investigated genotypes or LL and GM muscles (Table 2).

**Table 2:** The mean values of meat quality properties for different genotypes and muscles.

Item	PM		PMB		SE	Effect		
	LL	GM	LL	GM		M	G	M × G
pH24	5.51	5.56	5.53	5.51	0.03	ns	ns	ns
$L^{*}$	31.82${}^{\mathrm{AX}}$	34.32${}^{B}$	33.89${}^{\mathrm{aY}}$	35.41${}^{b}$	0.48	${}^{**}$	${}^{**}$	ns
$a^{*}$	11.56${}^{X}$	12.06	12.40${}^{Y}$	12.32	0.27	ns	${}^{**}$	ns
$b^{*}$	3.69${}^{\mathrm{AX}}$	2.55${}^{B}$	4.37${}^{\mathrm{AY}}$	2.66${}^{B}$	0.21	${}^{**}$	${}^{**}$	ns
$C^{*}$	12.16${}^{X}$	12.4	13.19${}^{Y}$	12.67	0.29	ns	${}^{**}$	ns
H*	17.54${}^{\mathrm{Ax}}$	11.50${}^{B}$	19.03${}^{\mathrm{Ay}}$	11.92${}^{B}$	0.72	${}^{**}$	${}^{*}$	ns
Expressed juice (cm${}^{2}$ g${}^{-1}$)	20.98${}^{X}$	21.05${}^{X}$	23.67${}^{Y}$	23.68${}^{Y}$	0.67	ns	${}^{**}$	ns

Abbreviations are as follows: PM – Polish Merino; PMB – Polish Merino × Berrichone du Cher; LL – *M. longissimus lumborum*; GM – *M. gluteus medius*; SE – standard error; M – muscle effect; G – genotype effect; M × G – interaction of muscle and genotype; ns – not significant. Different superscripts in the same row represent significant differences between individual muscles (within the genotype) (a, b P < 0.05; A, B P < 0.01). Different superscripts in the same row represent significant differences between genotypes (within individual muscles) (x, y P < 0.05; X, Y P < 0.01). ${}^{*}$ P < 0.05. ${}^{**}$ P < 0.01.

The parameters determining the meat colour were affected by both the animal's genotype and the type of muscle. Compared to LL, the GM muscle was characterized by a higher value (P < 0.05) of lightness $L^{*}$ and lower (P < 0.05) values of yellowness $b^{*}$ and $H^{*}$ (P < 0.05) both in PM and PMB lambs. The differences in meat colour between investigated genotypes were noted only with respect to the *longissimus lumborum* muscle. The LL muscle of PMB crossbreeds had a higher (P < 0.05) value of lightness $L^{*}$. The PMB lambs were also characterized by higher (P < 0.05) redness $a^{*}$ and yellowness $b^{*}$ of LL muscle. The chroma ($C^{*}$), which is a measure of colour intensity, as well as $H^{*}$, which determines the exact colour, were also affected by the animal's genotype (Table 2).

Analysing the expressed juice, it was found that the meat of PM lambs was characterized by a lower (P < 0.05) value of this parameter compared to meat of PMB lambs both in LL and GM muscles. There were no statistically significant differences in the value of this feature between the examined muscles within the genotype.

### 3.2 Chemical composition of meat

The results of chemical composition of LL and GM muscles of investigated genotypes are given in Table 3. There were no statistical differences between examined genotypes or muscle types regarding the content of moisture, fat, and crude protein.

**Table 3:** The mean values for meat chemical composition for different genotypes and muscles (%).

Item	PM		PMB		SE	Effect		
	LL	GM	LL	GM		M	G	M × G
Moisture	74.18	74.05	74.45	73.94	0.45	ns	ns	ns
Protein	21.02	21.17	20.76	21.00	0.16	ns	ns	ns
Fat	4.13	3.99	4.07	4.29	0.35	ns	ns	ns
Total collagen	1.39${}^{\mathrm{Ax}}$	1.22${}^{B}$	1.29${}^{\mathrm{Ay}}$	1.15${}^{B}$	0.03	${}^{**}$	${}^{*}$	ns

The meat from PM lambs was characterized by higher (P < 0.05) collagen content compared to PMB crossbreeds but only in relation to LL muscle (Table 2). While analysing the type of muscle, a higher content (P < 0.05) of collagen was found in LL muscle compared to GM in both PM and PMB lambs.

**Table 4:** The groups and chosen fatty acid for different genotypes and muscles (g 100 g${}^{-1}$ of total fatty acid).

Item	PM		PMB		SE	Effect		
	LL	GM	LL	GM		M	G	M × G
Σ SFA	48.35${}^{\mathrm{Ax}}$	46.38${}^{B}$	46.85${}^{y}$	47.52	0.47	${}^{**}$	${}^{*}$	${}^{*}$
Σ MUFA	46.08	46.50	46.64	45.72	0.37	ns	ns	ns
Σ PUFA	5.54${}^{A}$	6.66${}^{B}$	6.15	6.26	0.40	${}^{**}$	ns	ns
Σ n6	3.70${}^{a}$	4.56${}^{b}$	4.10	4.09	0.32	${}^{*}$	ns	ns
Σ n3	1.22${}^{a}$	1.37${}^{b}$	1.35	1.32	0.07	${}^{*}$	ns	ns
n6/n3	3.02	3.32	3.05	3.05	0.16	ns	ns	ns
C18:2c9t11(CLA)	0.45${}^{\mathrm{aX}}$	0.51${}^{b}$	0.52${}^{Y}$	0.63	0.05	${}^{*}$	${}^{**}$	ns

### 3.3 Fatty acid profile

The analysis of fatty acid composition did not show the impact of an animal's genotype on the share of acid groups, except for the sum of saturated fatty acid (SFA) (Table 4). The higher amount of SFA (P < 0.05) was found in the PM group of lambs compared to PMB.

The amount of the main isomer of linoleic acid C18:2 *cis*9, *trans*11 (CLA) was influenced by the lamb's genotype and muscle type. The higher content (P < 0.05) of CLA was found in LL muscle of PMB lambs (Table 4). The differences in the level of C18:2 *cis*9, *trans*11 between LL and GM muscles were found only within the PM genotype, where the GM showed the higher content (P < 0.05) of this isomer. The GM muscle was also characterized by a higher amount of a total polyunsaturated acids (PUFAs), as well as PUFA n-6 and PUFA n-3 (P < 0.05) (Table 4).

### 3.4 The concentration of taurine, carnosine, and L-carnitine

The investigated lamb genotypes had no effect on the content of taurine, carnosine, and L-carnitine in the meat (Table 5). In contrast, the LL and GM muscles within each genotype differed in terms of taurine, carnosine, and L-carnitine levels. The *gluteus medius* muscle was characterized by higher (P < 0.05) content of taurine, while in the *longissimus lumborum* muscle a higher amount (P < 0.05) of carnosine has been found. In turn, larger content (P < 0.05) of L-carnitine was found in the GM muscle but only within the PMB group (Table 5).

**Table 5:** The taurine, carnosine, and L-carnitine concentration for different genotypes and muscles (mg 100 g${}^{-1}$ of meat).

Item	PM		PMB		SE	Effect		
	LL	GM	LL	GM		M	G	M × G
Taurine	59.90${}^{A}$	75.54${}^{B}$	63.37${}^{A}$	74.66${}^{B}$	3.56	${}^{**}$	ns	ns
Carnosine	225.75${}^{A}$	204.33${}^{B}$	226.54${}^{A}$	208.22${}^{B}$	3.74	${}^{**}$	ns	ns
L-carnitine	164.90	167.89	165.42${}^{a}$	170.76${}^{b}$	1.54	${}^{*}$	ns	ns

## 4 Discussion

### 4.1 Physical characteristics of the meat

The ultimate meat pH measured 24 h after slaughter between 5.5 and 5.8 may be defined as acceptable range for sheep meat (Devine et al., 1993). According to this, the pH values measured in current study ranged from 5.51 to 5.56 may be considered to be in an acceptable range and showed that the glycolysis process proceeded correctly. Similar to the present study, no difference in the pH value was recorded by Purchas and Zou (2008) between *longissimus* and *infraspiratus* muscles in different breeds of cattle. In turn, Carson et al. (2001) indicated a higher pH in the lamb meat of Scottish Blackface compared to its crossbreeds with the Blueface Leicester.

Meat colour is one of the main characteristics taken into account by consumers to evaluate its quality and freshness (Khliji et al., 2010). In the present study, meat of PMB lambs had higher colour coordinates than those of PM lambs. The higher value of $b^{*}$ parameter was also found by Pereza-Mercado et al. (2010) in the meat of crossbred lambs of Polypay × Ramboulliet in comparison with the pure Pelibuey breed. In turn, Carson et al. (2001), in a study of the meat quality of six different lamb genotypes, found a lower value of $b^{*}$ parameter only for meat of Scottish Blackface compared to other groups. Differences in beef meat lightness have been found by Purchas and Zou (2008), where the meat of the Angus breed had the highest $L^{*}$ values, whilst crossbreeds of Charolaise × Hereford × Friesian had the lowest. However, Bianchi et al. (2006) did not find the influence of lamb's genotype on meat colour. In the research of Rant et al. (2019), conducted on the meat of Polish Merino ram lambs, differences in the parameters characterizing the colour of meat between examined LL and GM muscles have not been found, whereas Realini et al. (2013), analysing the muscle type and its morphological structure on pork meat quality, showed a lower value of $L^{*}$ and higher $a^{*}$ parameters for the *masseter* muscle compared to the *longissimus thoracis* and *semitendinous*. According to the authors, the differences in meat colour characteristics may be due to different muscle fibre composition. In *masseter* muscle, mainly oxidative and oxidative–glycolytic fibres were found, while *longissimus thoracis* contained the most white glycolytic fibres.

The analysis of expressed juice in the present study showed that the meat of crossbred lambs had less ability to hold its own water in comparison to pure merino lambs, while there were no differences between LL and GM muscles within both genotypes. Different results were obtained by Purchas and Zou (2008) when comparing the quality of two types of muscles of different beef genotypes. In the above-mentioned studies there were no differences between genotypes but statistically less desirable values of this parameter have been found in *infraspinatus* compared to *longissimus* muscle. The ability of the meat to hold water influences the changes in its content during storage or heat treatment. This parameter is determined by many interacting factors, such as the structure of the muscle itself, its physicochemical characteristics (especially pH), as well as the treatment of meat after slaughter (Huff-Lonergan and Lonergan, 2005). Therefore, the differences between genotypes in the present study were not associated with pH because it was similar, but this could have been due to the muscle tissue structure and the strength of proteins to binding water, which were better in merino lambs (Huff-Lonergan and Lonergan, 2005).

### 4.2 Chemical composition of meat

The content in the meat of components such as protein, intramuscular fat, or water determine its nutritional value. In the conducted study, the level of these components in individual muscles within investigated genotypes was at a similar level. The differences in protein content have also not been found in studies of breast and thigh muscles of different hen lines (Intarapichet and Maikhunthod, 2005). In turn, in the study of Radzik-Rant et al. (2014), a higher content of intramuscular fat was found in LL muscle of dual-purpose wool–meat type lambs than in the Polish Heath breed, which is characterized by poor meat performance.

Similar to the present study, the muscle type (*longissimus dorsi, semitendinosus, biceps femoris, semimembranous*) did not affect the content of water, protein, and intramuscular fat in Galician Blonde cattle (Ruiz et al., 2010). Ablikim et al. (2016) also reported no differences in fat content between LL and GM muscles in Chinese sheep breeds. In turn, Esenbuga et al. (2009) showed that the muscle type did not affect the intramuscular fat content, whereas it influences the contents of protein and moisture in Awassi lambs. The muscle type also had an effect on moisture, protein, and intramuscular fat in pork meat (Realini et al., 2013). The highest level of protein and lowest of intramuscular fat was determined in *longissimus thoracis* muscle compared to *semitendinous* and *masseter*.

In the present study the differences in the content of collagen were found both between genotypes and muscle types. The obtained results are in agreement with these of Pereza-Mercado et al. (2010), who noted the influence of animal breed on the collagen content in meat. Brzostowski et al. (2006) also obtained the greater share of collagen in the *longissimus dorsi* muscle of Pomeranian sheep compared to its crossbreeds with Blackface meat breed. Considering the effect of muscle type on the collagen content Tschirhart-Hoelscher et al. (2006) determined the higher level of it in *gluteus medius* than in *longissimus dorsi* muscle, contrary to the present study. Similarly, Realini et al. (2013) showed the lowest amount of collagen in *longissimus thoracis* muscle in pork. However, Ablikim et al. (2016) confirmed a higher content of this component in LL muscle compared to GM.

### 4.3 Fatty acid profile

In the present study the animal genotype showed the impact on the share of the sum of saturated fatty acid, wherein the meat of PM lambs had the higher amount of SFA compared to PMB. These findings were in line with study of Radzik-Rant et al. (2014) conducted on two diverse sheep breeds, who stated the higher share of SFA in the meat of Polish Lowland lambs bred for the same purpose as the Polish Merino breed, compared to the primitive Polish Heath sheep, which was characterized by the lower intramuscular fat content. In the present study, a smaller amount of intramuscular fat was determined in LL muscle of PMB lambs, which may indicate a greater share of phospholipids that are a component of cell membranes in the structure of which unsaturated and especially polyunsaturated acids (PUFA) are incorporated (Wood et al., 2008). It may explain the slightly higher share of PUFAs in LL muscle in PMB lambs.

When the effect of genotype on the content of the main isomer of linoleic acid C18:2 *cis*9, *trans*11 (CLA) was examined, a significantly higher amount of this compound was found in LL muscle of PMB group. In the study of Purchas and Zou (2008), a higher content of CLA was also detected in *longissimus thoracis* and *infraspinatus* muscles of bovine crossbreeds compared to pure breeds. In the present study the GM muscle was characterized by the significantly higher level of CLA, as well as total PUFAs, PUFA n-6, and PUFA n-3. The higher content of PUFA n-6 in GM muscle confirmed the previous results obtained by Janovska et al. (2010), indicating the greater capacity of the accumulation of this acid family by muscles with a higher proportion of oxidative fibres, which may be more in GM muscle than in LL. The differences in the content of fatty acids between various muscle types were indicated by Purchas and Zou (2008) in cattle and De Brito et al. (2017) in White Dorper lambs.

### 4.4 The concentration of taurine, carnosine, and L-carnitine

The conducted study showed that the genotype of the animals did not affect the level of taurine, carnosine and L-carnitine, while differences in the level of these components were noted between the investigated muscle types. The differences in the content of taurine and carnosine depending on muscle type have been noted in other studies. Purchas et al. (2004) recorded the lower amount of taurine and higher amount of carnosine in LL muscle compared to GM in the Texel crossbreed lambs and in comparison with *semitendinosus* and *triceps brachii* muscles in the pure Romney lambs. The higher content of taurine and lower amount of carnosine in the muscles with a greater proportion of oxidative and oxidative–glycolytic fibres like *masseter*, *infraspinatus*, *semitendinous*, and even *gluteus medius* were recorded in cattle and pork meat (Dunnett and Harris, 1997; Aristoy and Toldra, 1998; Purchas and Zou, 2008). The higher level of carnosine in muscles like *longissimus* with a higher proportion of glycolytic fibres results from the buffering capacity of this dipeptide, which allows for maintaining the acid–base homeostasis. Thus, the concentration of carnosine would be higher in glycolytic muscles that are more susceptible to acidification and have higher buffering capacity compared to oxidative muscles (Artioli et al., 2010).

The amount of L-carnitine in meat due to its association with oxygen metabolism may also depend on the type of muscle and the proportion of different fibres in it. Muscles with a greater content of oxidative and oxidative–glycolytic fibres may be characterized by a higher content of L-carnitine. Shimada et al. (2004), examining the concentration of L-carnitine in meat of various animal species, showed its greater share in red muscle (*m. soleus*) compared to white muscle (*m. pectoralis profundus*) in laying hens. The red oxidative muscles have low glycogen but high lipid concentrations, where L-carnitine may be involved in energy production. This may explain the higher content of this compound in GM muscle in the conducted research.

In the present study, the genotype had no effect on the content of analysed bioactive components in the lamb meat. Similarly, in the study of Purchas and Zou (2008), the differences in the amount of carnosine and taurine between beef genotypes were minor, although a greater impact of the breed was noted for taurine. A higher content of this component was found in the groups of crossbreeds compared to the pure breeds, whereas Intarapichet and Maikhunthod (2005) found statistical differences in carnosine not only between white and red broiler muscles but also between genotypes for analysed individual muscles.

## 5 Conclusions

The obtained results showed a greater impact of the lamb's genotype on the physical characteristics of meat, apart from the pH value, than on its chemical composition and the content of bioactive components. The muscle type had an effect on meat colour; collagen content; fatty acid profile; and the amount of taurine, carnosine, and L-carnitine.

The meat of Polish Merino lambs was brighter in colour and had more favourable value of expressed juice. In terms of the content of basic components, the investigated genotypes differed only in the content of collagen, the amount of which was higher in the *longissimus lumborum* muscle of Polish Merino lambs. In turn, the meat of Polish Merino × Berrichone du Cher crossbreeds contained less SFA but a higher amount of bioactive CLA.

The differences between the investigated *longissimus lumborum* and *gluteus medius* muscles concerned meat colour; collagen content; fatty acid profile; and the amount of taurine, carnosine, and L-carnitine. The *gluteus medius* muscle was lighter in colour and had the lower collagen content. This muscle was characterized by the higher PUFA content from both the n-6 and n-3 family and a greater share of the C18:2 *cis*9, *trans*11 CLA isomer. The *gluteus medius* also contained more taurine and L-carnitine, while the carnosine content was significantly higher in the *longissimus lumborum* muscle.

## Data Availability

The data are available from the corresponding author upon request.
